# Supplementary material for: Consequences of removal of exotic species (eucalyptus) on carbon and nitrogen cycles in the soil-plant system in a secondary tropical Atlantic forest in Brazil with a dual-isotope approach
Source: PeerJ. 2020 May 28;8:e9222. doi: 10.7717/peerj.9222 (PMC7261475; doi:10.7717/peerj.9222)

**DataS1**

Images of União Biological Reserve (ReBioUnião), Brazil. (A) secondary forest; (B) site managed 12 months ago (M12); (C) site managed 3 months ago (M3).


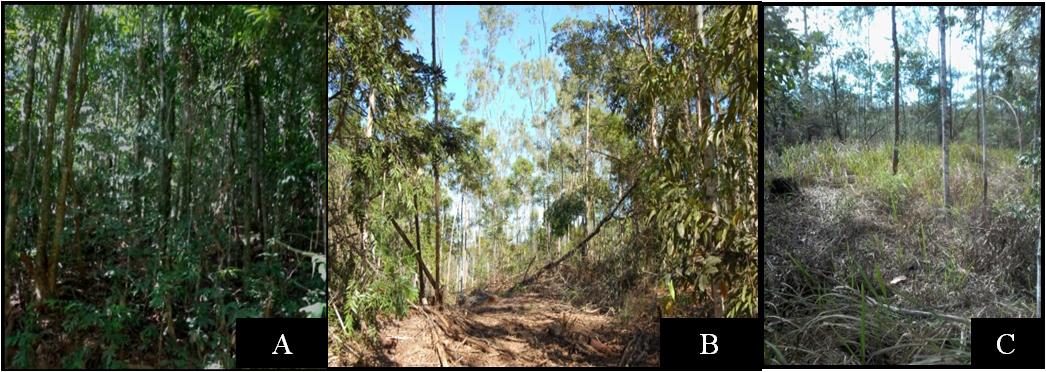

Supplement: Supplemental Information 1 [file peerj-08-9222-s001.docx]
