# Supplementary material for: Consequences of removal of exotic species (eucalyptus) on carbon and nitrogen cycles in the soil-plant system in a secondary tropical Atlantic forest in Brazil with a dual-isotope approach
Source: PeerJ. 2020 May 28;8:e9222. doi: 10.7717/peerj.9222 (PMC7261475; doi:10.7717/peerj.9222)
Supplement: Supplemental Information 2 — Family and species sampled in the secondary forest of União Biological Reserve (Rebio União), Brazil. [file peerj-08-9222-s002.doc]

**DataS2**

Family and species sampled in the secondary forest of União Biological Reserve (ReBioUnião), Brazil.

| Family/Species |
| --- |
| Annonaceae  *Xylopia sericea* A. St.-Hill  Apocynaceae  *Geissospermum laeve* (Vell.) Miers. |
| Bignoniaceae  *Handroanthus chrysotrichus* (Mart. ex DC.) Mattos. |
| Euphorbiaceae |
| *Mabea fistulifera*Mart. |
| *Senefeldera verticillata* (Vell.) Croizat |
| Fabaceae  *Apuleia leiocarpa* (Vogel) J.F. Macbr. |
| *Pseudopiptadenia contorta* (DC.) G.P. Lewis & M.P. Lima |
| Lauraceae  *Ocotea diospyrifolia* (Meisn.) Mez |
| Melastomataceae |
| *Miconia hypoleuca* (Benth.) Triana |
| *Tibouchina estrellensis* (Raddi) Cogn.  Malpighiaceae  *Byrsonima sericea* D.C. |
| Meliaceae  *Guarea guidonia* (L.) Sleumer |
| Moraceae  *Brosimum glazioui* Taub. |
| *Ficus gomelleira* Kunth & C.D. Bouché |
| *Helicostylis tomentosa* (Poepp. &Endl.) Rusby |
| Myristicaceae  *Virola bicuhyba* (Schott ex Spreng.) Warb. |
| *Virola gardneri* (A. DC.) Warb. |
| Sapindaceae  *Cupania racemosa* (Vell.) Radlk. |
| Sapotaceae |
| *Micropholis guyanensis* (A. DC.) Pierre |
| Siparunaceae  *Siparuna guianensis* Aubl. |
