# Supplementary material for: Consequences of removal of exotic species (eucalyptus) on carbon and nitrogen cycles in the soil-plant system in a secondary tropical Atlantic forest in Brazil with a dual-isotope approach
Source: PeerJ. 2020 May 28;8:e9222. doi: 10.7717/peerj.9222 (PMC7261475; doi:10.7717/peerj.9222)
Supplement: Supplemental Information 3 [file peerj-08-9222-s003.docx]

**DataS3**

Medium diameter (µm) of reference certified samples for laser diffraction particle analyzer (SALD-3101, Shimadzu).

| Certified sample | 75% diameter  (µm) | 50% diameter  (µm) | 25% diameter  (µm) |
| --- | --- | --- | --- |
| JIS 11 | 4,17 ± 0,99 | 2,16 ± 0,41 | 0,94 ± 0,27 |
| Licopodium | 34,09 ± 1,74 | 31,57 ± 1,56 | 28,82 ± 1,41 |
| glass beads | 1490 ± 94 | 1355 ± 90 | 1221 ± 84 |
